# Vitamin A: too good to be bad?

**DOI:** 10.3389/fphar.2023.1186336

**Published:** 2023-05-22

**Authors:** Guoxun Chen, Sabine Weiskirchen, Ralf Weiskirchen

**Affiliations:** ^1^ College of Food Science and Technology, College of Biomedicine and Health, Huazhong Agricultural University, Wuhan, China; ^2^ Institute of Molecular Pathobiochemistry, Experimental Gene Therapy and Clinical Chemistry (IFMPEGKC), RWTH University Hospital Aachen, Aachen, Germany

**Keywords:** retinol, vitamin, liver, hepatic stellate cell, hypervitaminosis A, hypovitaminosis A

## Abstract

Vitamin A is a micronutrient important for vision, cell growth, reproduction and immunity. Both deficiency and excess consuming of vitamin A cause severe health consequences. Although discovered as the first lipophilic vitamin already more than a century ago and the definition of precise biological roles of vitamin A in the setting of health and disease, there are still many unresolved issues related to that vitamin. Prototypically, the liver that plays a key role in the storage, metabolism and homeostasis of vitamin A critically responds to the vitamin A status. Acute and chronic excess vitamin A is associated with liver damage and fibrosis, while also hypovitaminosis A is associated with alterations in liver morphology and function. Hepatic stellate cells are the main storage site of vitamin A. These cells have multiple physiological roles from balancing retinol content of the body to mediating inflammatory responses in the liver. Strikingly, different animal disease models also respond to vitamin A statuses differently or even opposing. In this review, we discuss some of these controversial issues in understanding vitamin A biology. More studies of the interactions of vitamin A with animal genomes and epigenetic settings are anticipated in the future.

## 1 Introduction

**FIGURE 1:**
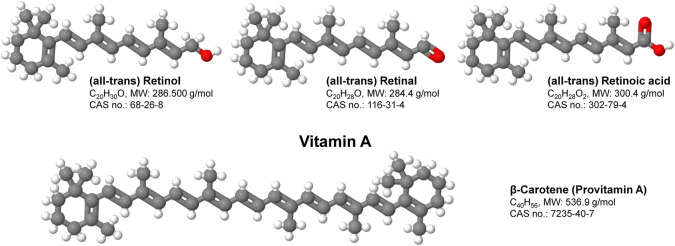
Structure and function of vitamin A. Vitamin A is a group of fat-soluble, oxygen and UV-sensitive compounds that include retinol, retinal, retinoic acid and several provitamin A carotenoids such as β-Carotene. Vitamin A has multiple functions within the body including the promotion of development and growth and support of the immune system. Shortcomings in Vitamin A are majorly associated with disorders of vision, skin, bone and the immune system.

Vitamin A (VA, *all-trans*-retinol) is an essential nutrient and called preformed VA, which it can be processed to form retinal, and retinoic acid (RA). VA can come from provitamin carotenoids (i.e., β-carotene and other carotenoids) (Figure 1). These key micronutrients play important roles in vision, reproductive biology, immune function, bone remodeling and epithelial tissue homeostasis (Haaker et al., 2020). The activity of VA was discovered as the first lipophilic vitamin more than a century ago (McCollum and Davis, 1913). Since then, retinol and its metabolites have been demonstrated to regulate various physiological processes (Ross and Harrison, 2007). Dietary VA and molecules with its activities have been gradually identified as reviewed elsewhere (Ross, 2003; Blomhoff and Blomhoff, 2006; Ross and Harrison, 2007; Napoli, 2012). Both the changes of VA status and activation of VA signaling have been shown to regulate a variety of physiological pathways including metabolism (Chen and Chen, 2014; Blaner, 2019a). Interestingly, changing VA statuses (feeding animals diets with different amounts of VA) and activation of VA signaling (treating animals and humans with pharmacological compounds such as 13-cis retinoic acid) sometimes do not obtain results that fit each other well. In addition, data obtained from rat and mouse studies do not align well. This mini review was aimed to discuss these discrepancies and challenges in VA studies.

**FIGURE 2:**
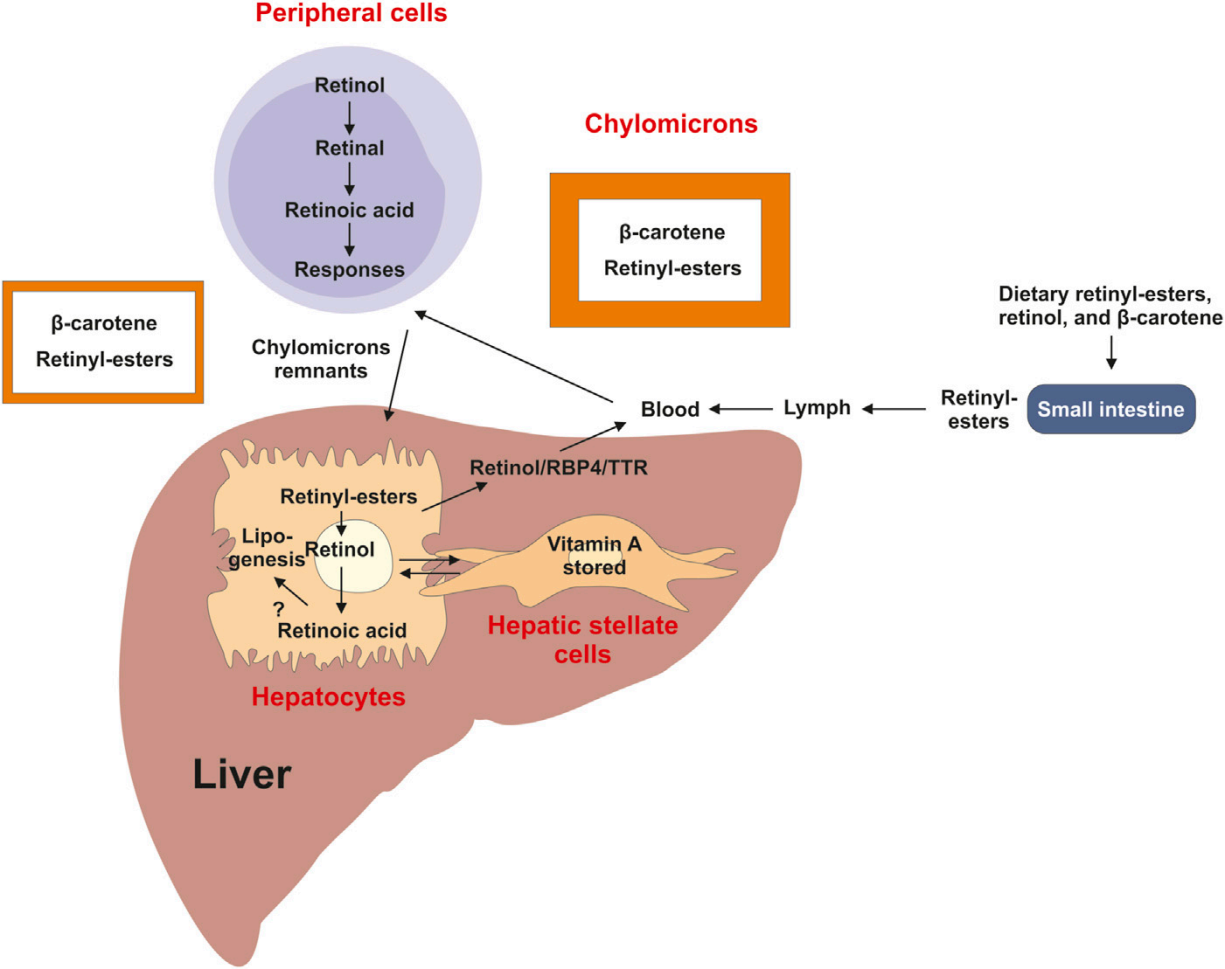
Overview of vitamin A uptake, metabolism and transport. Dietary retinyl-esters and carotenoids are absorbed by enterocytes of the intestine lumen, esterified and packed into chylomicrons. These large lipoprotein complexes are then secreted into the circulation, processed into chylomicron remnants, which are then readily taken up by the liver and peripheral cells and tissues. In the liver most of the vitamin A is stored as retinyl-esters in hepatic stellate cells. This store can be mobilized when needed back to retinol by hydrolysis, which can be transported in complex with retinol binding protein 4 (RBP4) and transthyretin (TTR) via the circulation to target organs. A directional transport distributes retinol between hepatic stellate cells and hepatocytes, in which retinoic acid is the main driver of lipogenesis.

As a micronutrient, VA contributes to the general health of humans (Bloomhoff and Blomhoff, 2006). As humans cannot synthesize it, VA has to be derived from diets such as liver, fish, milk, eggs, and orange or yellow vegetables. Dietary molecules with VA activities exist in two forms, the preformed VA and provitamin A (Ross et al., 2020). Preformed VA molecules are retinol and retinyl-esters that are generally obtained from animal derived foods, whereas provitamin A carotenoids are from plant-derived foods. Retinyl-esters as the major stored form of VA are digested into retinol and fatty acids in the gastrointestinal tract, and absorbed into enterocytes. Carotenoids in enterocytes and hepatocytes mainly in the form of β-carotene are enzymatically converted into retinaldehyde, which is reduced to produce retinol for further process (Lakshman, 2004; Wyss, 2004). Retinol is esterified with fatty acids to generate retinyl-esters, which are incorporated into chylomicrons and transported in the lymph and peripheral cells and transported via the blood into the liver, in which retinol circulates between hepatocytes and hepatic stellate cells (HSCs) (Figure 2).

The hepatic retinyl-ester content is the best indicator of VA status as VA is mainly stored in the liver (Tanumihardjo, 2004). The Food and Nutrition Board at the Institute of Medicine (IOM) of the National Academies has established the dietary reference intakes (DRIs) of VA (Medicine Io, 2001). According to age and gender, the recommended dietary allowance (RDA) of VA is given in μg. Since 2021, the IOM has recommended to use retinol activity equivalent (RAE), in which 1 µg of RAE is equivalent to 1 µg of retinol, 2 µg of supplemental β-carotene, 12 µg of dietary β-carotene or 24 µg of dietary α-carotene or β-cryptoxanthin (Institute of Medicine, 2001). The RDA for VA is 900, 700, and 770 μg of RAE for men, women, and pregnant women at age 19–50 years old (Institute of Medicine, 2001). Unfortunately, other studies still use International Units (IUs) for reporting VA intakes, recommendations, or allowances, which often results in confusion (Dwyer et al., 2020). To convert IUs into µg RAE, the following conversion factors should be applied (National Institutes of Health, 2022):

1 IU retinol = 0.3 µg RAE; 1 IU supplemental β-carotene = 0.3 µg RAE; 1 IU dietary β-carotene = 0.05 RAE.

Nevertheless, irrespectively in which VA quantities are given, both, VA deficiency (VAD) and excessive intake of VA have severe health consequences (Table 1). The differences of β-carotene equivalence of VA in the recommended absolute values among institutes and in units of expression of VA have been noticed (Melse-Boonstra et al., 2017). Although worldwide the VA RDAs are largely within the same ranges for men, women and children, global recommendations for dietary intake may differ from country to country due to the differences of β-carotene conversion rate. Furthermore, the biological effects of insufficient or excess supply with vitamin A can be significantly different between animals and humans.

**TABLE 1:** Health risks and signs resulting from Vitamin A deficiency and excess intake of Vitamin A^a^.

Primary and secondary vitamin A deficiency (hypovitaminosis A)	Acute and chronic vitamin A poising (hypervitaminosis A)
Loss of appetite	Severe headache (increased intracranial pressure)
Growth retardation	Blurred vision
Keratinization of epithelial cells and poor wound healing	Nausea and vomiting
Nervous disorders	Dizziness
Defective reproduction	Aching muscles and abdominal pain
Fetal malformations (e.g., lungs)	Coordination problems
Night blindness, dry eyes, and xerophthalmia (i.e., drying and clouding of the cornea)	Increased spinal fluid pressure
Increased susceptibility for infections and acne	Congenital birth defects and malfunctions of the eye, skull, lungs and heart
Disorders in bone formation	Potential teratogenic effects
Increased risk for cysts formation in endocrine glands	Reduced bone mineral density
Higher prevalence for formation urinary calculi and nephritis	Drowsiness and irritability
Increased risk of anemia and death	Portal hypertension and hepatic fibrosis
Infertility	Hair loss, cracked lips and dry skin
Large number of secondary complications	Elevation of blood calcium levels

^a^ Data was compiled among others from (LiverTox, 2012; Harrison EH, 2014; NIH, 2022).

Provitamin A, β-carotene is enzymatically cleaved to produce retinaldehyde, which is reduced to generate retinol (O’Byrne and Blaner, 2013). In the small intestine, retinyl-ester is hydrolyzed into retinol and a fatty acid by lipases (Harrison, 2005). Provitamin A molecules can be found in colored fruits and vegetables such as β-carotene, α-carotene, and β-cryptoxanthine. Two enzymes, annotated as β-carotene-dioxygenase-1 and -2 (BCO1 and BCO2), are key enzymes in β-carotene metabolism to VA (Lakshman, 2004; Wyss, 2004; von Lintig et al., 2020). BCO1 mediates the central cleavage across the C15,C15′ double bond adjacent to a canonical β-ionone ring site of carotenoids and β-apocarotenoids (von Lintig et al., 2020). In the case of β-carotene this results in the formation of two retinal molecules. BCO2 has a broader substrate specificity and catalyzes the asymmetric oxidative cleavage of carotenoids across the C9,C10′ double bond, yielding apo-10′-carotenals and ionones whose functions are still uncertain (von Lintig et al., 2020; Strychalski et al., 2022). Therefore, BCO1, the central cleaving β-carotene-15,15′-dioxygenase, is considered the only VA producing enzyme.

**FIGURE 3:**
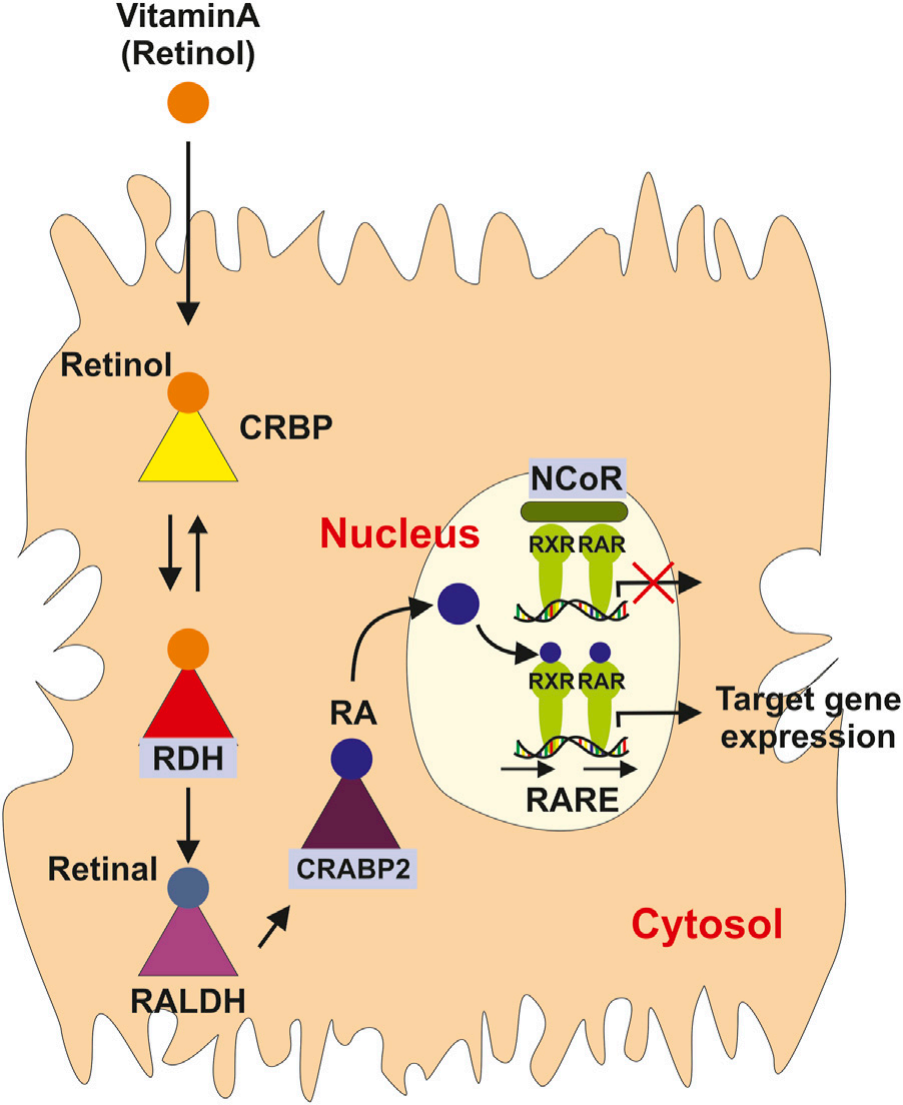
Simplified vitamin A signaling. In the cytoplasm, vitamin A is bound to the cellular retinol-binding protein (CRBP), where it is oxidized in a two-step reaction involving a retinol dehydrogenase (RDH) and a retinal dehydrogenase (RALDH) into retinoic acid (RA). RA is then transported from the cytosol to the nucleus by bind to the cellular retinoic acid binding protein type II (CRABP2) to the nucleus where it bind to members of the nuclear retinoic acid receptors (RAR) that form heterodimers with the retinoid X receptor (RXR) family members that subsequently bind to specific retinoic acid response elements (RARE), thereby initiating the transcription of target genes. In the absence of the RA ligand, RAR/RXR heterodimers bound to DNA are associated with nuclear co-repressors (NCoR) that repress gene expression.

In the body, the blood VA level reflects the dynamic balance of the dietary uptake, storage and usage in organs and tissues. The blood VA levels declines when severe VA deficiency occurs. Many enzymes and proteins involved in VA absorption, transport, and metabolism have been identified and their corresponding genes have been cloned (Moise et al., 2007). So far, retinal and RA are the two metabolites mediating VA’s physiological functions (Ross, 2003; Evans and Mangelsdorf, 2014). Retinal is the chromophore of visual pigments, a critical component of the visual cycle. On the other hand, RA is an activator of nuclear receptors, which are transcription factors responding to variations of ligand levels. There are about 48 members in the nuclear receptor super family (Evans and Mangelsdorf, 2014). Their ligands are a variety of molecules from steroid hormones, thyroid hormones, vitamin D, oxysterols, bile acids, RA, and many others (Evans and Mangelsdorf, 2014). The structural features of nuclear receptors allow the binding of the ligands to initiate transcriptional regulations to influence physiological responses (Bain et al., 2007). Additional cofactors interacting with ligand-activated nuclear receptors modulate the target gene expressions (Benbrook et al., 2014; Evans and Mangelsdorf, 2014). As an agonist, RA activates RA receptors (RARα, *β*, *γ*), and retinoid X receptors (RXRα, *β*, *γ*) (Wolf, 2010; Naploi, 2012). Here, RXRs can interact with other nuclear receptors to form a heterodimer to regulate gene expression, and 9-*cis* RA has been thought to activate RXRs (Figure 3) (Evans and Mangelsdorf, 2014). The RA response elements (RAREs) are DNA fragments located on the promoters of RA responsive genes composed classically of two direct repeats of a consensus sequence 5-RGKTCA-3’ (‘R’ = purine, ‘K’ for “keto” = ‘G’ or ‘T’, according to International Union of Biochemistry and Molecular Biology rules) separated by one, two or five nucleotides (Moutier et al., 2012). Other nuclear receptors, such as the hepatocyte nuclear factor 4α (HNF4α), chicken ovalbumin upstream promoter-transcription factor II (COUP-TFII), and peroxisome proliferator-activated receptor β/δ (PPARβ/γ) have also been considered to mediate RA signaling (Makita et al., 2001; Shaw et al., 2003; Kruse et al., 2008; Li et al., 2014).

## 2 Effects of vitamin A status on the regulation of glucose and lipid metabolism

Numerous studies have indicated that VA has a role in metabolic disease prevention and causation. It is required for maintaining development, normal growth, immunity, reproduction, and vision. There are additional functions and there is growing evidence that RA and some of its metabolites are important transcriptional regulators, which mediate their effects by members of nuclear hormone superfamily of ligand-dependent transcriptions factors (for review see Blaner, 2019b). More recent studies have shown that VA and related proteins involved in its metabolism have further roles in the development and prevention of obesity and diseases related to obesity such as insulin resistance, type 2 diabetes, hepatic steatosis, steatohepatitis, and cardiovascular disease (Blaner, 2019b). Some of the important findings in humans and animals should be highlighted in the following paragraphs.

### 2.1 The effects of changes of VA status on humans

Based on the data obtained in a meta-analysis including 54 studies, the prevalence of VAD in Chinese children is about 5.16% with inequities between rural and urban areas (Song et al., 2017). One study has investigated the micronutrients statuses and intakes in women of reproductive age and during pregnancy in Ethiopia, Kenya, Nigeria and South Africa (Harika et al., 2017). The deficiencies in VA, iodine, zinc and folate ranged from 4% to 22%, 22%–55%, 34% and 46%, respectively, which are associated with inadequate intakes of these micronutrients in those women (Harika et al., 2017). To combat VAD, direct supplementation and food fortification of retinyl-esters (Sommer, 2008), and agriculturally-enriched food containing β-carotene (Tang et al., 2009) have been used. Recently, orange-fleshed or golden sweet potatoes that contain a significant amount of β-carotene and other micronutrients have been considered a potential way to reduce malnutrition in VAD regions (Gurmu et al., 2014). These are already low-priced crops and staple foods in Africa and can be easily applied to other places for the intervention of VAD. Interventions using orange sweet potato showed various results, which seem to depend on the length of the study, gender and region (Haskell et al., 2004; van Jaarsveld et al., 2005; Lowe et al., 2007; Hotz et al., 2012a; Hotz et al., 2012b; Jamil et al., 2012; Turner et al., 2013; Girard et al., 2017). For children and women, this orange color potato seems to be sufficient to increase the intakes of VA equivalent (β-carotene) and lead to the improvement of plasma VA levels (van Haarsveld et al., 2005; Low et al., 2007; Hotz et al., 2012a; Hotz et al., 2012b; Girard et al., 2017). On the other hand, it only works for men (Haskell et al., 2004), but not women (Jamil et al., 2012; Turner et al., 2013) of Bangladeshi in studies covering 60 days or shorter. It appears that even if provitamin A is provided in certain forms, it does not mean the plasma retinol will be raised, indicating that more studies are needed to determine the conversion of β-carotene to retinol in humans.

One Brazilian study analyzed the VA status in severely obese adolescents (64 subjects, 15–19 years old) before and 1, 6 and 12 months after the Roux-en-Y gastric bypass procedure (Silva et al., 2017). VAD was observed in 26.6% of the subjects before the procedure, and blood retinol levels dropped 1 month after the procedure. A daily uptake of 5000 IU retinyl-acetate, corresponding to 1,500 μg VA, failed to reverse VAD and night blindness, suggesting the importance of dietary VA. This is associated with reductions of blood glucose and lipid levels (Silva et al., 2017). Low VA was positively associated with high lipids, inflammation and insulin resistance in obese Mexico children (García et al., 2013). A French study showed that in Caucasian outpatients (267 subjects, aged 40.5 ± 12.6 years), lower values of VA are associated with increasing body mass index (Takahashi et al., 2021). The blood level of glycated hemoglobin has a weak inverse correlation with VA in subjects with type 2 diabetes mellitus (T2DM) (Maxwell et al., 1997). The plasma RA levels of uncontrolled T2DM subjects and control subjects were compared using a sensitive liquid chromatography–tandem mass spectrometry method (Morgenstern et al., 2021). It appears that RA levels in T2DM subjects are lower than that in those control ones. However, the plasma retinol levels of those subjects were not reported (Morgenstern et al., 2021).

On the other hand, an Indian study that enrolled 146 subjects reported no difference in VA status between normal weight and obese women (Herter-Aeberli et al., 2016). An Iranian study showed that VA intake was not different from the recommended dietary values in morbid obese subjects prior to bariatric surgery (170 subjects, mean age 37 years old) (Malek et al., 2019). In the Canadian population, the intake and plasma levels of VA in subjects with diabetes are similar to that in the control subjects (Basualdo et al., 1997). Contrarily, a Japanese study containing 197 subjects with T2DM did not find any relationship between VA intake and loss of muscle mass (Takahashi et al., 2021).

In elderly Chinese (>64 years), an increase in blood VA level as measured by a LC-MS/MS method was associated with increases in alanine aminotransferase, glutamyl transpeptidase, urea, glucose, and uric acid levels (Yin et al., 2021). A cross sectional study (1,928 children, aged 7–11 years) performed in Chongqing (China) showed that the blood VA levels in obese subjects are higher than that in overweight and normal weight subjects, and glucose level is strongly associated with VA status (Wei et al., 2016). Moreover, in Brazilian adolescents, elevated blood retinol level and reduced β-carotene levels were associated with dyslipidemia (Albuquerque et al., 2016). As the blood VA level is not a good indicator for the VA status, it is probably premature to say that the elevated blood VA level indicates a healthy state. The results from clinical observations appear to indicate that more studies are needed to fully understand the complex roles of VA in the regulation of glucose and lipid metabolism of different human populations.

### 2.2 The effects of changes of VA status in animals

When the Bio-Breed (BB)/Worcester (Wor) diabetic-prone diabetic rats, a model of type 1 diabetes mellitus (T1DM) were fed a diet enriched in VA and zinc, they had a higher rate of developing diabetes than those fed the basal diet or the diet only supplemented with VA (Lu et al., 2000). In addition, an inbred strain of BB rats, VAD leads to reduction of T1DM and insulitis in inbred BB diabetes-prone Wor rats (Driscoll et al., 1996). In male albino rats fed a diet enriched in retinyl palmitate (30,000 IU per day) for 2 days, the total hepatic triglycerides and cholesterol, glycogen and VA contents were elevated (Singh et al., 1969). This was associated with increases in the hepatic gluconeogenesis and glycogen (Dileepan et al., 1977). The adrenals appear to contribute to glycogen and gluconeogenesis associated with hypervitaminosis A (Singh et al., 1975; Singh et al., 1976a; Singh et al., 1976b). In addition, the glucose tolerance was lowered in rats fed excessive amount of VA (Singh et al., 1968). Furthermore, excessive VA uptake has been thought to contribute to fatty liver development in rats (Singh et al., 1969).

Interestingly, VA supplementation was reported to correct the hyperglycemia and hyperinsulinemia, but not to increase body weight, in female rats (8–10 weeks of age) fed an high fat diet for 6 weeks (El-Sayed et al., 2013). Dietary supplementation of 129 mg retinyl palmitate/kg diet for 14 weeks led to elevated liver triacylglycerol and plasma insulin levels, and lowered plasma glucose level in 30-week old WNIN/GR-Ob obese rats compared with that of 2.6 mg retinyl palmitate/kg diet (Jeyakumar et al., 2017). The treatment with a VA-enriched diet leads to body weight gain in lean and WNIN/GR-Ob obese rats (Jeyakumar et al., 2015).

On the other hand, lowered dietary VA status corrected obesity and T2DM development in Zucker diabetic fatty (ZDF) rats (Wang et al., 2022). In addition, feeding of a VAD diet prevented the development of obesity in Zucker fatty rats (Zhang et al., 2012). In fact, VAD in rats had led to a reduction of plasma glucose and lipid levels (Zhang et al., 2012). Interestingly, in lecithin-retinol acyltransferase (*Lrat* ^−/−^) null, but not the wild type, mice fed a VA-deficient diet for 10 weeks, blood glucose was elevated and glucose tolerance test results were impaired according to a study (Trasino et al., 2015). Although the *Lrat* ^−/−^ mice has reduction of pancreatic retinol, the retinol content of the liver and other impacts of the lack in LRAT on glucose metabolism were not analyzed (Trasino et al., 2015). Nevertheless, the discrepancy between mouse and rat responses to VAD is worth to be investigated further.

## 3 Effects of retinoids on the regulation of glucose and lipid metabolism

RA is a drug to treat human diseases. Isotretinoin (13-*cis* RA) has been used to treat acne, but causes side effects such as hyperlipidemia and reduction of insulin sensitivity in human subjects (Koistinen et al., 2001), which was attributed to *de novo* lipogenesis (Laker et al., 1987). In line, sensitive subjects are more likely to develop hyperlipidemia and metabolic syndrome later (4 years after the completion of the isotretinoin therapy) (Rodondi et al., 2002). In addition, hyperlipidemia was also observed as a side effect of RA treatment in patients with adult T cell leukemia (Maeda et al., 2008), and acute promyelocytic leukemia (Fujiwara et al., 1995).

RA (10 mg RA/kg body weight per day) has been used to treat both Zucker lean (ZL) and ZDF rats at 9 weeks of age for 2 weeks (Nizamutdinova et al., 2013). This treatment lowered the blood glucose level of ZDF rats from 528 to 223 mg/dL, which was attributed to the inhibition of NF-κB signaling and reduction of inflammation (Nizamutdinova et al., 2013). However, this study did not report the blood glucose measurements in ZL rats (Nizamutdinova et al., 2013). Female *ob/ob* mice at 9 weeks of age had been treated with 100 µg/mouse/day of RA or vesicle for 16 days (Manolescu et al., 2010). This RA treatment lowered body weight, and improved outcomes of intraperitoneal glucose and insulin tolerance tests (Manolescu et al., 2010). In obese mice induced by feeding a high-fat/high-sucrose diet for 16 weeks, RA treatment reduced body weight, hyperlipidemia, and improved insulin responses, which was attributed to the activations of PPARβ/δ and RAR (Berry and Noy, 2009). In diet-induced obese and *ob/ob* mice, RA treatment for 7 days was reported to ameliorate hepatic steatosis (Kim et al., 2014).

Here, the results of human and mouse studies do not fit well with each other. In subjects with acne and leukemia, RA treatment leads to hyperlipidemia and reduction of insulin sensitivity. It should be noted that 13*-cis*-RA is generally used to treat acne, whereas rodents were treated with all-*trans*-RA. This may contribute to the different study outcomes. On the other hand, in rodents, RA treatment appears to improve insulin responses and correct obesity and hyperlipidemia. Whether this discrepancy is due to the different pharmacological responses of humans and mice to RA or RA signaling is mediated by different proteins in humans and rodents remains to be determined. Nevertheless, it clearly demonstrates the challenges in the journey to understand the mechanisms by which RA signaling regulates glucose and lipid metabolism.

## 4 The vitamin A metabolism in stellate cells and hepatocytes

**FIGURE 4:**
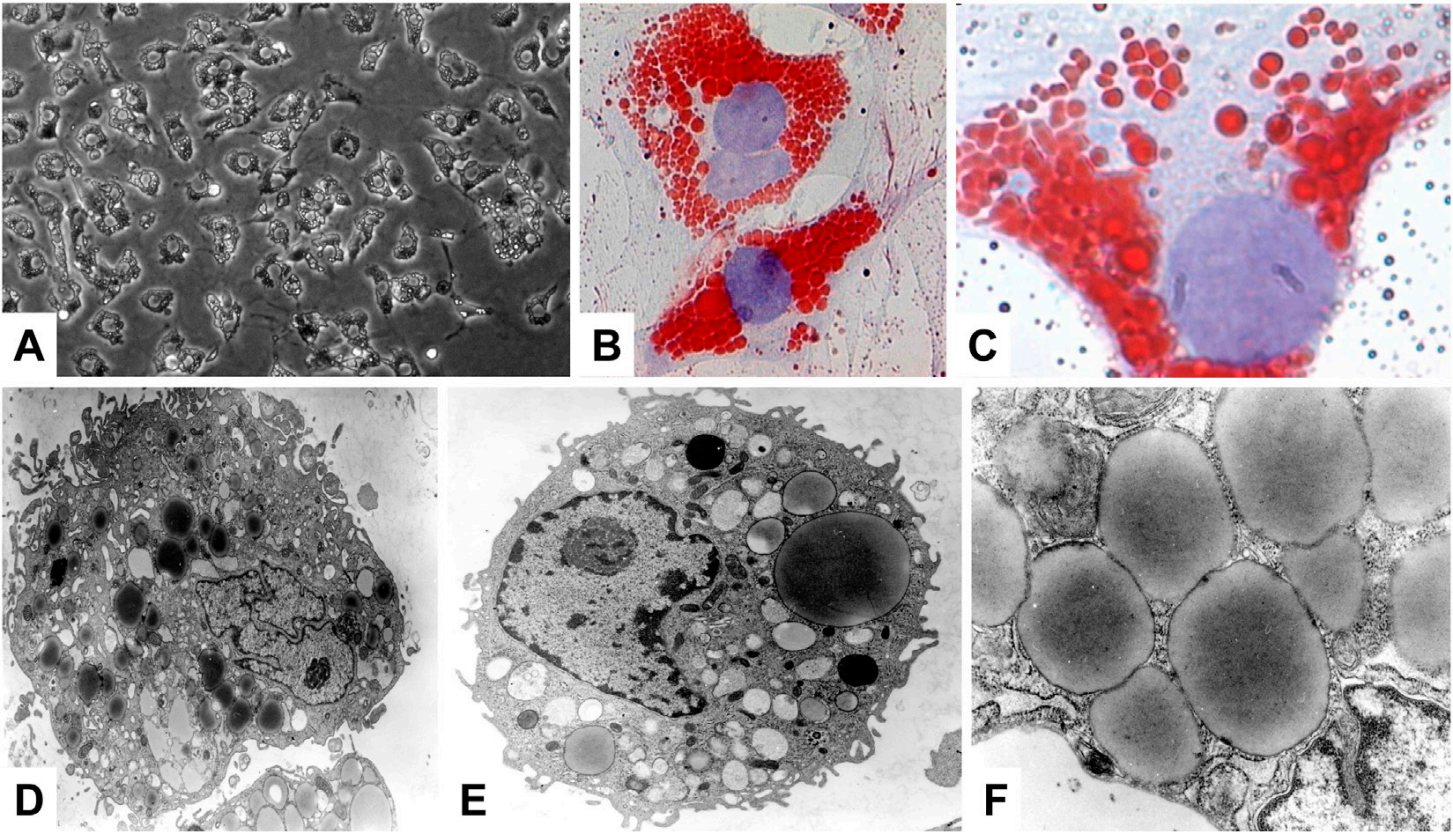
Hepatic stellate cells, major store for retinoids. **(A)** Phase contrast microscopic analysis of primary rat HSC. **(B,C)** Oil Red O staining was used to visualize the cytoplasmic lipid droplet content of HSC. **(D–F)** Representative electron microscopy of primary HSCs cultured for 1 day. Ultrastructural characteristics of HSCs include the occurrence of abundant, size variable vitamin A-containing droplets, a prominent nucleus, and numerous cytoplasmic extensions. Original magnifications are **(A)** ×200, **(B)** ×400, **(C)** ×1,000, **(D)** ×6,500, **(E)** ×8,300, and **(F)** ×30,000, respectively. All images were reprinted with permission from (Neyzen et al., 2006).

Most of the VA (80%–90%) is stored in the liver, while only a small portion is stored in extrahepatic organs such as the pancreas, lungs, kidneys and intestines (Senoo et al., 2010). In the liver, the major storage side for VA are HSCs that were formerly called as VA-storing cells, lipocytes, pericytes, or fat storing cells. These are located in the space of Disse between hepatocytes and sinusoidal endothelial cells (Senoo et al., 2010). In respective cells, VA is stored in large cytoplasmic lipid-droplets of different sizes that can be effectively stained with Oil Red O (Figure 4). The content of VA within HSCs has a gradual distribution in the liver lobules and depends on the total VA amount and is further genetically determined (Weiskirchen and Tacke, 2014).

**FIGURE 5:**
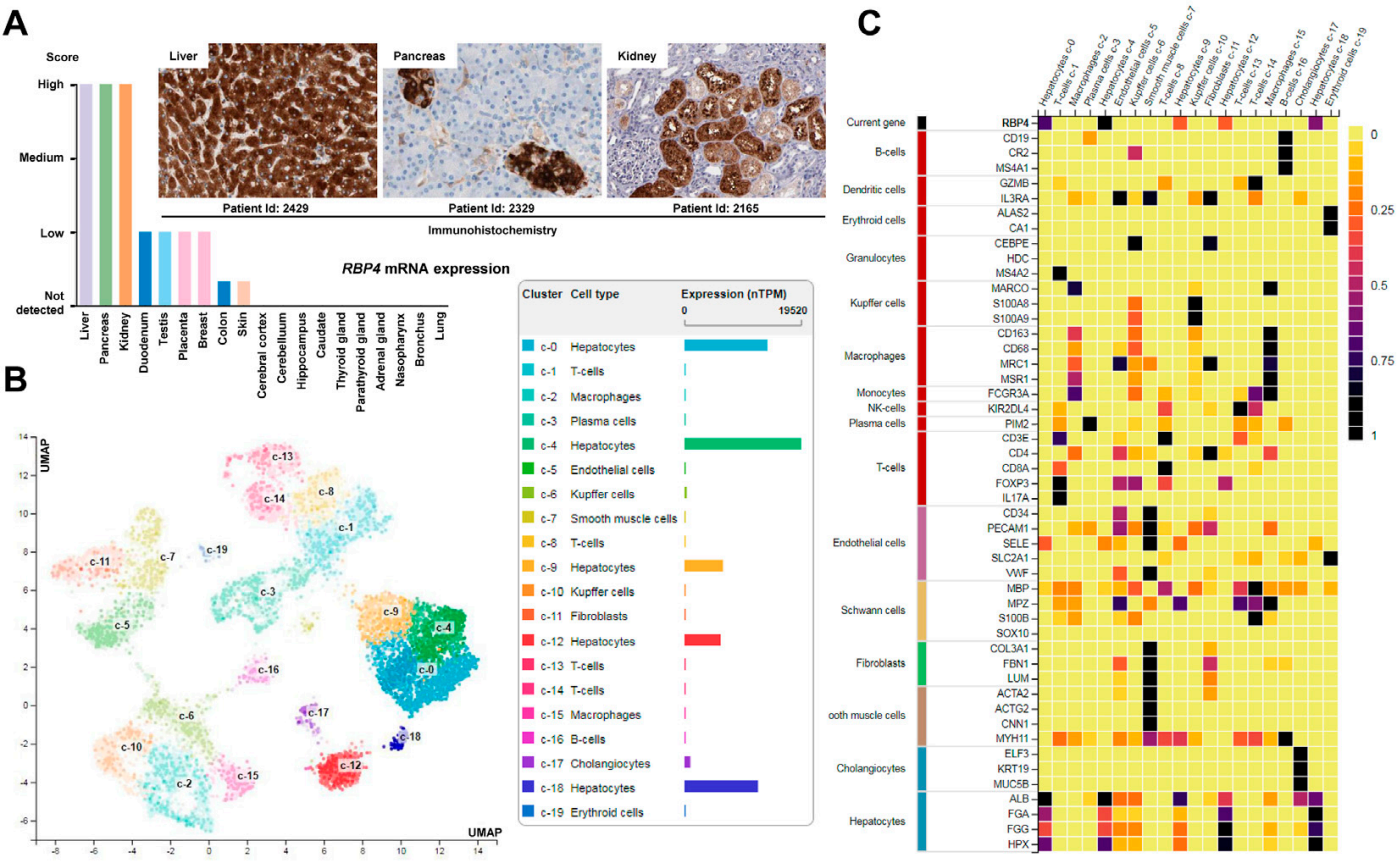
Expression of RBP4. **(A)** Expression of RBP4 in healthy human tissue. Highest expression of RBP4 is found in the liver, pancreas and kidney. Immunohistochemistry shows that the major cellular sources of RBP4 in the liver are hepatocytes, while in pancreas the highest expression is found in endocrine cells (islet) and in the kidney in renal proximal tubules. **(B)** Single cell expression data of liver cells confirms that the highest expression of RBP4 in liver is found in hepatocytes. **(C)** The expression of RBP4 in liver correlates with expression of albumin (ALB), fibrinogen α polypeptide chain (FGA), fibrinogen γ polypeptide chain (FGG), and hemopexin (HPX) that are well-established markers of hepatocytes (Naylor et al., 1987; Fish and Neerman-Arbez, 2012; Gu et al., 2017). All data depicted in this figure is taken from the Human Protein Atlas resource (https://www.proteinatlas.org/).

Actually, the precise role of the stored hepatic retinoids for the cellular function of HSCs is not fully understood. Dietary retinol that is, taken up from the circulation by hepatocytes can be transferred to HSCs, where it is converted by the LRAT to retinyl-esters and stored in lipid droplets. Upon retinol shortage, these retinyl-esters can be utilized and transferred back to hepatocytes (Haaker et al., 2020). The bidirectional transport of retinyl-esters is strongly dependent on retinol-binding proteins (RBPs). In particular, RBP4 seems to have key functions. In the human body, RBP4 is highly expressed in the liver, in renal proximal tubules, pancreatic endocrine cells (i.e., the so called pancreatic islets), and in some other tissues (Figure 5A). In the normal liver, the highest expression is found in hepatocytes, while the expression in non-parenchymal cells (Kupffer cells, smooth muscle cells) and fibroblasts is rather low (Figure 5B). Therefore, it is often used as a hepatocyte marker, that is co-expressed with typical hepatocytic genes including albumin (ALB), fibrinogen α polypeptide chain (FGA), fibrinogen γ polypeptide chain (FGG), and hemopexin (HPX) (Figure 5C). Experimental findings demonstrated that antibodies directed against RBP4 can completely block the transport of retinol from hepatocytes to HSCs (Bloomhoff et al., 1988). Therefore, it was first postulated that HSCs take up and accumulate retinol and RBP4 in the liver from a retinol-RBP4 complex making this RBP central for the storage of retinol in the liver (Bloomhoff et al., 1988). However, this view was somewhat challenged by the finding that *Rbp4*-deficient C57BL/6 mice accumulate similar amounts of retinyl-esters in the liver combined with serum retinol levels that were below the detection limit, suggesting that the absence of RBP4 does not impair accumulation of hepatic retinol stores (Shen et al., 2016). Similar findings were already reported by Quadro et al. (2004) several years ago showing that RBP4 is not involved in the intercellular trafficking of retinol between hepatocytes and HSCs in two mouse models, namely, in *Rbp4* null mice and a transgenic mouse that expressed human RBP4 under the control of the mouse muscle creatine kinase promoter in the RBP4 null background. The roles of RBP1 and RBP4 in the transfer of retinoids from hepatocytes to HSCs have been reviewed (Blaner et al., 2016). It appears that the identities of the molecules facilitating retinoid movement from hepatocytes to HSCs remain to be revealed (Widjaja-Adhi and Golczak, 2020).

**FIGURE 6:**
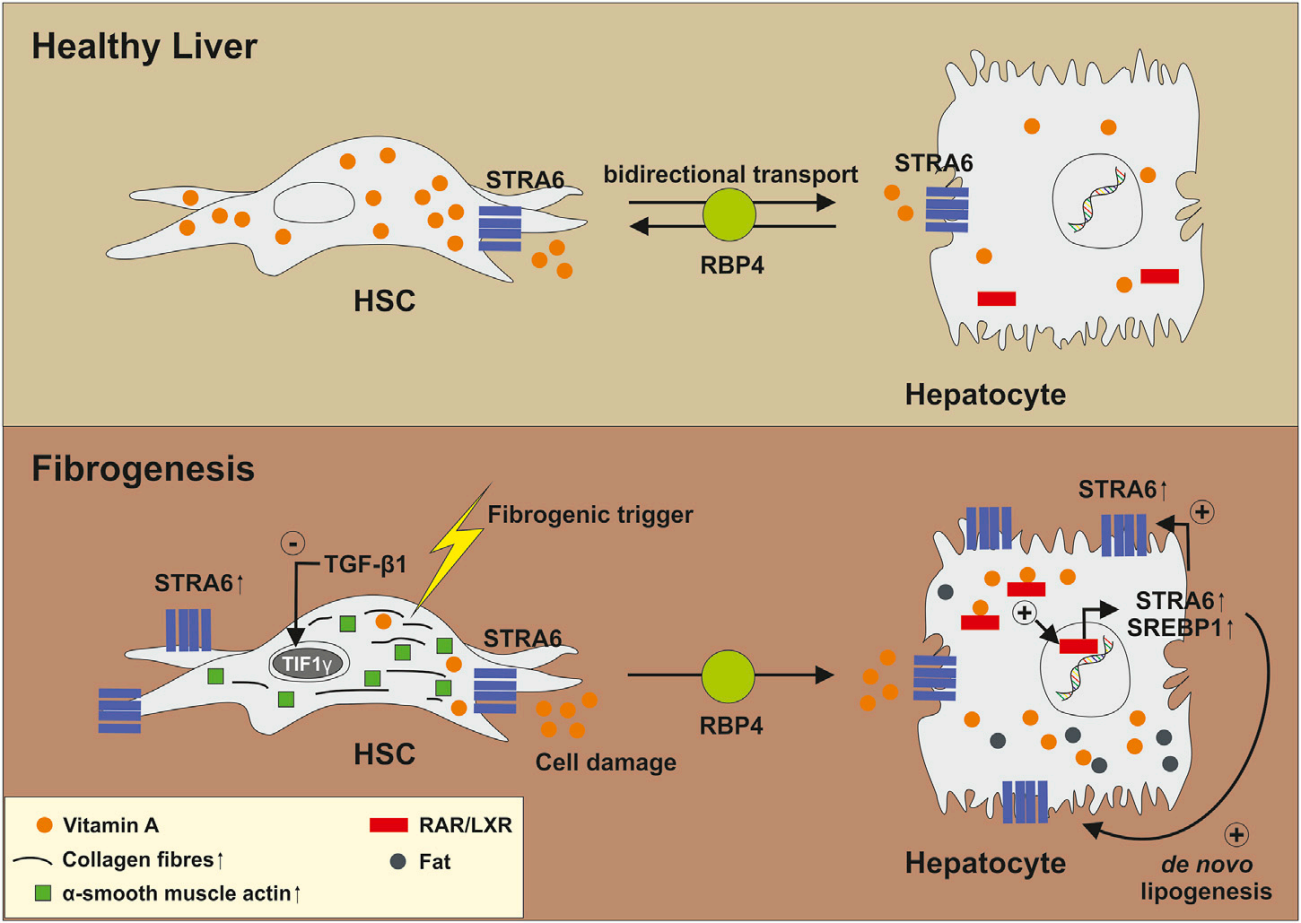
Biological function of STRA6 in the pathogenesis of liver fibrosis. In healthy liver, STRA6 bidirectional transports Vitamin A between extra- and intracellular retinoid binding proteins and acts as a *bona fide* RBP. Fibrogenic triggers induce expression of TGF-β1 and leakage of HSCs. This results in suppression of TIF1γ and increased expression of STRA6. Released Vitamin A is transported to and taken up by surrounding hepatocytes. This results in a virtuous cycle in which STRA6 expression is increasingly expressed. Moreover, genes involved in *de novo* lipogenesis are induced via RAR/LXR-mediated signalling resulting in accumulation of fat. For more information, please refer to the text.

More recent data suggested that retinol released from HSCs has paracrine effects on hepatocytes by promoting influx and accumulation of triglycerides as well as increasing the expression of genes associated with *de novo* lipogenesis (Hwang et al., 2021). This activity is of fundamental importance during activation of HSCs, which is the key step in the initiation of hepatic fibrosis. It was postulated that in the first of liver fibrosis, TGF-β1 suppresses the transcriptional intermediary factor 1γ (TIF1γ) in activated HSCs that subsequently increases expression of the stimulated by retinoic acid 6 (STRA6) gene (Lee et al., 2020; Hwang et al., 2021). STRA6 is a channel-like transmembrane pore, which bidirectional transports VA between extra- and intracellular RBPs and acts itself as a *bona fide* RBP (Kelly and von Lintig, 2015). High expression of STRA6 in HSCs leads to a leakage of retinol from HSCs that is, taken up by surrounding hepatocytes through STRA6. This leads to transcriptional activation of SREBP1 and further induction of STRA6 through RAR/LXR, which in consequence induces a vicious cycle in hepatocytes resulting in fat deposition and increased expression of genes associated with lipogenesis (Figure 6) (Hwang et al., 2021). However, these findings were not reproduced yet by any other laboratory and more studies are needed to confirm this line of research.

Nevertheless, the general role of STRA6 in liver is still completely unknown. Under normal conditions, hepatic STRA6 expression in mouse adult liver is rather low and the complete lack of STRA6 in mice majorly affects the quantities of VA in the eye, while there were no phenotypic alterations observed (Bouillet et al., 1997; Ruiz et al., 2012; Terra et al., 2013; Amengual et al., 2014). Moreover, *Stra6* ^−/−^ mice displayed higher hepatic retinoid stores than wild type mice when kept on a beta-carotene diet, while there was no increase in hepatic VA when fed with a VA-sufficient diet (Moon et al., 2022). A similar finding was previously found in a study in which *STRA6* ^−/−^ mice were kept on a standard chow on which *Stra6* ^−/−^ showed 6-fold higher quantities of hepatic retinyl-ester levels than age-matched heterozygous siblings (Kelly et al., 2016). In contrast, high *STRA6* expression is found in the brain, kidney spleen, female genital tracts, and testis (Bouillet et al., 1997). Importantly, elevated expression of *STRA6* was demonstrated in blood-organ barriers made of epithelial or endothelial cells associated with tight junctions. Therefore, it was originally suggested that STRA6 performs a function as a transport system, which is in agreement with the proposed activity in VA transport (Bouillet et al., 1997).

So there seems to be a complex network of factors (RBPs, STRA6, RAR/LXR, TIF1γ, and others) that impact the transfer of VA compounds from HSCs to hepatocyte and vice versa leaving open many unanswered questions about all of these compounds. Moreover, high blood levels of RBP4 are associated with the pathogenesis of metabolic disease, obesity, insulin resistance, and T2DM (Flores-Cortez et al., 2022) showing that components of this network have more complex activities than simply controlling or transferring VA compounds between different cells.

Nevertheless, deficiency or impairment in RA metabolism worsens inflammation, fibrosis progression, and accelerates tumorigenesis in the liver (Weiskirchen and Tacke, 2014; Okayasu et al., 2019). In this context it is notable that hepatic damage is associated with activation of HSCs that lose their VA stores and transit into α-smooth muscle actin positive, extracellular matrix-secreting myofibroblasts (MFBs) that have the capacity to synthesize large quantities of extracellular matrix, which is the major hallmark of hepatic fibrogenesis. Although mostly argued that “HSC activation is simply accompanied by loss of the HSC typical retinoid droplets,” the biological function of retinoid loss in HSC biology and in hepatic fibrosis is still unclear (Kitto and Henderson, 2020). Recently, it was suggested that the cellular release of active VA metabolites, such as RA, is an essential event that is, required to initiate the differentiation of the Th17 cell population, thereby limiting the secretion of IL-17A by immune cells and promoting IL-22 signalling that shows protective effects in mouse models of liver pathology, but only in the absence of IL-17A (Rolla et al., 2016; Kartasheva-Ebertz et al., 2021). Therefore, the release of VA during activation of HSCs might be a prerequisite, consequence or even protective defence mechanism during the pathogenesis of hepatic lesions.

Another unsolved issue is the mechanisms by which elevated VA concentrations lead to hepatotoxicity that can arise acutely after consumption of exorbitant quantities of performed VA, or chronically after the persistent intake of lesser, but still excessive amounts of preformed VA (Harrison, 2014). In particular, the finding that elevated stores of VA in HSCs can provoke dose related cellular activation, hypertrophy and increased expression and excess collagen production in humans is hard to understand (Nollevaux et al., 2006; LiverTox, 2012). It was speculated that the excess of VA *per se* is potentially not the major cause of VA-induced fibrosis sickening effects of VA. Instead metabolites of VA with or without the influence of other environmental factors may give rise to polar metabolites, which may in turn induce liver cell necrosis and inflammation because of their covalent binding to hepatocellular proteins (Nollevaux et al., 2006). Dominantly activating VA signalling through the expression of a constitutive active RAR in mouse liver recaps the phenotype (Blaner, 2019b). In this scenario, HSC activation and fibrogenesis is the net result of retinol toxicity and release of pro-inflammatory and pro-fibrotic mediators by damaged hepatocytes. Similar to humans, the excessive intake of VA was associated with HSC lipidosis and hepatic fibrosis in a domestic cat that was fed exclusively on a home-made diet based on raw beef liver and giblets highly enriched in VA (Guerra et al., 2014).

However, if excess VA induces hepatic fibrosis, it is somewhat questionable why arctic animals such as polar bears or bearded seals that have similar HSC content in % of parenchymal cells but much higher VA-storing capacity in HSCs than humans do not show signs of hypervitaminosis A or spontaneous hepatic fibrosis (Senoo et al., 2012). Moreover, it is still open how and why arctic animals accumulate such high quantities of VA. Recently it was demonstrated in rats that the repeated daily intake of VA (90 µg/100 g body weight) for longer than 14 days formed resistance to hypervitaminosis A that prevented the accumulation of more than 250–300 µg VA/g liver tissue and decreased the content of vitamins E and C (Bozhkov et al., 2022). It is worth to note that LRAT knock-out mice had reduced hepatic VA storage which probably has increased hepatic RA levels (Liu and Gudas, 2005). Interestingly, animals with Cu-induced liver fibrosis were unable to accumulate VA within their liver, further suggesting that the functional state of the liver impacts the general capacity of the liver to uptake VA (Bozhkov et al., 2022). Alternatively, the storage of VA might be a mechanism to counteract excessive supply.

Similarly, the association of primary (i.e., prolonged dietary deprivation) and the secondary (i.e., shortages in absorption, storage, or transport of VA) VAD and the outcome of hepatic fibrosis is not fully understood. It is well accepted that VAD is associated with liver disease progression. In particular, it is assumed that poor VA intake leads to oxidative stress that critically contributes to the pathophysiological mechanism during non-alcoholic fatty liver disease (NAFLD), the severity of chronic liver diseases, and hepatocellular carcinoma (Coelho et al., 2020). However, a case-control study that enrolled 60 healthy and NAFLD patients from Jordanian showed that the intake of VA in both groups was not significantly different (734.9 ± 46.5 vs. 895.3 ± 54.5, *p* = 0071) suggesting that poor uptake might not *per se* be necessarily be a prerequisite for initiating the NAFLD pathogenesis (Tayyem et al., 2019). Furthermore, VA restriction in male rats induced by feeding a VA-deficient diet for 3 months resulted in a hypolipidaemic effect by decreasing serum triacylglycerol, cholesterol and HDL-cholesterol levels. This is contrarily to the common findings associated with NAFLD, in which *de novo* lipogenesis, triglyceride accumulation, and elevated serum cholesterol quantities elevated are hallmarks of this disease (Oliveros et al., 2007). Even more, an experimental study that analysed VA metabolism in two mouse models of NAFLD showed a significant accumulation of VA in hepatocytes, suggesting that impaired VA metabolism is the consequence but not the reason for NAFLD pathogenesis (Saeed et al., 2021). These discrepancies show that VA might act in concert with other factors that together predict the pathogenesis and outcome of NAFLD. Possibly, these factors are genetically determined or other nutrient components (Obrochta et al., 2014).

In sum, all these findings demonstrate that there are still many unanswered questions related to the mechanisms by which high quantities of VA mediate its pathogenic effects during acute and chronic hypervitaminosis A.

## 5 Challenges and future perspectives

Although VA was discovered already more than a century ago and its metabolism extensively studied, there is still a significant lack in understanding of its biological activities. As an essential micronutrient, it is required for the maintenance of normal vision and is indispensable for proper immune function, bone remodeling, epithelial tissue homeostasis and functionality of the reproductive system. Different organizations and countries recommend minimum and maximum values for VA intake for children, adults, men, and normal women, pregnant and lactating women. Based on the important functions of VA and diseases that are directly or indirectly connected with VA signaling pathways, this vitamin is intensively discussed as a therapeutic compound to cure various diseases (Chen et al., 2022; Cook et al., 2022; Sajovic et al., 2022; Zawada et al., 2023). In liver disease, for example, a low circulating retinol level was associated with liver fibrosis and liver-related mortality in chronic liver disease, suggesting that supplementation with VA might have direct therapeutic implications (Song and Jiang, 2023). However, different VA compounds are taken up at different ratios and both, shortage and oversupply, cause severe clinical symptoms. Unfortunately, the outcome of some experimental studies and clinical trials are contradictory, most likely because the biological activities of VA compounds are influenced by partly unknown genetic and epigenetic factors. Therefore, the research area of VA’s role in hepatic metabolism and energy homeostasis still offers many open questions. In particular, there is a lack of knowledge in the following areas:

• How can somebody estimate his/her daily VA intake when different VA compounds are absorbed differentially in the body (e.g., the absorbance rate of retinoids is 75%–100%, while the uptake of carotenoids is strongly dependent on the food matrix and type of carotenoid)?
• Why do humans and animals (e.g., artic animals) are differentially susceptible for hypervitaminosis A?
• How can discrepancies be explained that were shown in different correlation studies of body mass index and VA status?
• What are the mechanisms that lead to liver damage in hyper- and hypovitaminosis A?
• How specific is the VA status for the pathogenesis of liver diseases?
• What are suitable tests for estimating VAD/excess?
• Which genetic and epigenetic factors in other pathways impact uptake, storage, metabolism and elimination of VA?
• How suitable are experimental models of VAD/excess for mimicking human disease conditions?
• What are normal VA values in the body and which diagnostic tests are suitable for measuring them?
• Are there test systems available that allow to detect VAD/excess at an early stage?
• Is there a reasonable composition of foodstuffs that guarantees an adequate VA supply?
• Can the knowledge about VA transport lead to novel therapeutic opportunities (e.g., in NASH)?

Importantly, there are many studies that have associated VAD or excess with a multitude of symptoms and diseases. In addition, VA is a well-accepted epigenetic modulator (Bar-El Dadon and Reifen, 2017). However, the molecular mechanisms by which VA exerts its effect on the expression of genes or signatures are not fully understood. Similarly, the network of VA, RAR/RXR, and diverse nuclear co-repressors (e.g., NCoRs) is not unraveled yet. The beneficial effects of VA in the treatment of skin diseases, cystic acne, wound healing injuries, and several cancers have shown that this vitamin is an effective therapeutic. Nevertheless, over dosage can cause severe side effects including respiratory distress, pericardial effusions, and multi-organ failure. Therefore, the intake of VA supplements or diets enriched in VA should be only done after consulting an expert.

## Abbreviations

DRI(s), dietary reference intake(s); HSC(s), hepatic stellate cell(s); LRAT, lecithin-retinol acyltransferase; MFB, myofibroblast(s); NAFLD, non-alcoholic fatty liver disease; PPAR, proliferator-activated receptor; RA, retinoic acid; RARE(s), retinoic acid response element(s); RBS(s), retinol binding protein(s); RDA, recommended dietary allowance; RXR, retinoid X receptors; STRA6, stimulated by retinoic acid 6; T1DM, type I diabetes mellitus; T2DM, type II diabetes mellitus; TIF1γ, transcriptional intermediary factor 1γ; VA, vitamin A; VAD, vitamin A deficiency; ZDF, Zucker diabetic fatty.
